# Genetics, Synergists, and Age Affect Insecticide Sensitivity of the Honey Bee, *Apis mellifera*

**DOI:** 10.1371/journal.pone.0139841

**Published:** 2015-10-02

**Authors:** Frank D. Rinkevich, Joseph W. Margotta, Jean M. Pittman, Robert G. Danka, Matthew R. Tarver, James A. Ottea, Kristen B. Healy

**Affiliations:** 1 Department of Entomology, Life Sciences Annex, Louisiana State University Agricultural Center, Baton Rouge, LA, United States of America; 2 USDA-ARS Honey Bee Breeding, Genetics, and Physiology Laboratory, Baton Rouge, LA, United States of America; University of North Carolina, Greensboro, UNITED STATES

## Abstract

The number of honey bee colonies in the United States has declined to half of its peak level in the 1940s, and colonies lost over the winter have reached levels that are becoming economically unstable. While the causes of these losses are numerous and the interaction between them is very complex, the role of insecticides has garnered much attention. As a result, there is a need to better understand the risk of insecticides to bees, leading to more studies on both toxicity and exposure. While much research has been conducted on insecticides and bees, there have been very limited studies to elucidate the role that bee genotype and age has on the toxicity of these insecticides. The goal of this study was to determine if there are differences in insecticide sensitivity between honey bees of different genetic backgrounds (Carniolan, Italian, and Russian stocks) and assess if insecticide sensitivity varies with age. We found that Italian bees were the most sensitive of these stocks to insecticides, but variation was largely dependent on the class of insecticide tested. There were almost no differences in organophosphate bioassays between honey bee stocks (<1-fold), moderate differences in pyrethroid bioassays (1.5 to 3-fold), and dramatic differences in neonicotinoid bioassays (3.4 to 33.3-fold). Synergism bioassays with piperonyl butoxide, amitraz, and coumaphos showed increased phenothrin sensitivity in all stocks and also demonstrated further physiological differences between stocks. In addition, as bees aged, the sensitivity to phenothrin significantly decreased, but the sensitivity to naled significantly increased. These results demonstrate the variation arising from the genetic background and physiological transitions in honey bees as they age. This information can be used to determine risk assessment, as well as establishing baseline data for future comparisons to explain the variation in toxicity differences for honey bees reported in the literature.

## Introduction

The number of colonies of commercially kept honey bees, *Apis mellifera* L., in the United States has been reduced by one-half since the 1940s [1, 2]. Contemporary overwintering losses average nearly 30%, which is double the rate that is suggested as being economically acceptable [3]. The decline of honey bees and other pollinators is occurring when the proportion of crops requiring insect pollination and the value of those services is increasing at a rate outstripping supply [1, 4]. Therefore, identifying factors causing honey bee declines is an important research goal.

The decline of honey bee colonies in the United States is very complex and driven by biologic, abiotic, economic, and political forces [5]. One of the most concerning factors is exposure of honey bees to insecticides. Sources of insecticide exposure include agricultural practices, residential and urban pest control, aerial or truck-mounted applications targeting public health pests, and in-hive miticide treatments. More than 120 pesticides have been found in samples of wax, pollen, and bees in colonies from the US and Canada with an average of 6 pesticides detected in each colony [6]. Further complicating the impact of insecticides are in-hive miticides applied by beekeepers that may interact synergistically with other pesticides [7, 8]. These synergistic interactions are often overlooked as much of the pesticide exposure to honey bees is often at doses or concentrations lower than the LD_50_/LC_50_ value of each individual pesticide. Commercial beekeepers (>500 colonies, 88% of all colonies surveyed) are much more likely to implicate colony losses due to pesticide exposure than small scale beekeepers likely due to the exposure to pesticides of bees in agricultural operations where pollination services are provided [3]. Therefore, identifying the impacts of pesticides on honey bees is concerning to large commercial bee keeping operations.

Several insecticides have received recent attention, and emphasis has been placed on evaluating the impact of these pesticides on bees. Of particular concern are the neonicotinoids that are widely used as seed treatments in industrial agriculture. At sublethal doses, neonicotinoids, such as imidacloprid and thiamethoxam, can reduce colony health [9], impair foraging [10], and disrupt learning and memory [11]. To combat the rise of mosquito-vectored pathogens such as the West Nile and Chikungunya viruses, mosquito control professionals routinely utilize insecticides such as naled (an organophosphate) or resmethrin (a pyrethroid) to reduce mosquito populations [12, 13]. Many pyrethroid containing mosquito control products (i.e. Duet®, AquaAnvil®, et al.) contain piperonyl butoxide (PBO) which synergizes pyrethroid toxicity by inhibiting cytochrome P450 monooxygenases [14]. Naled has adverse impacts on honey bees, especially when applied while bees are outside the hive during foraging and night time bearding [15]. Beekeepers use coumaphos and amitraz in colonies to control populations of the parasitic Varroa mite (*Varroa destructor*). While coumaphos and amitraz are not acutely toxic to honey bees, they do have the potential to synergize interactions with other insecticides and fungicides [7, 8].

Honey bees are a eusocial species in which the division of labor is largely dictated by age [16, 17]. As bees age and transition from young, in-hive workers to foragers, their physiology, including metabolic, neurologic, and muscular processes, is immensely modified [18, 19]. Physiological differences have also been observed among stocks of honey bee with different genetic backgrounds [20, 21]. However, little evidence exists on how age and genetic background affect the sensitivity of honey bees to insecticides. Understanding these physiological differences between stocks and ages of bees is crucial to accurately determining the effects of insecticides on honey bees and extrapolating that information into best management practices and policy.

## Materials and Methods

### Honey Bees for Bioassays of Genetic Background and Synergism

Honey bees were reared according to standard beekeeping practices but without miticides, antibiotics, or supplemental feeding at the USDA-ARS Honey Bee Breeding, Genetics, and Physiology Laboratory in Baton Rouge, LA. Bees were allowed to forage freely on naturally occurring vegetation in the surrounding area. Each colony was headed by a naturally mated queen. Queens of commercially available Italian (*A*. *mellifera ligustica*) and Carniolan (*A*. *mellifera carnica*) stocks were established in colony divisions in the spring of 2014 (Italian Bees: Wooten’s Golden Queens, Palo Cedro, CA; Carniolan Bees: Strachan Apiaries, Yuba City, CA). Russian bees, a stock derived from selection within a closed breeding population, were from colonies maintained at the USDA laboratory.

To test for differences between stocks of honey bees in insecticide sensitivity and synergistic interactions, brood combs with emerging adult bees were collected from each colony between April and August and held at 33 ± 0.1°C with 70 ± 5% humidity in a dark incubator. One-day-old bees were brushed from the combs and sorted into groups of 20 into 475-ml wax cups (Innopack, Orange, CA) and then weighed. The cups had holes punched in the bottom for ventilation and were covered with nylon tulle secured with rubber bands. Bees were held at environmental conditions listed above with 3 cotton balls saturated with a 50% (w/v) sucrose solution until bees were three days of age. Insecticide toxicity and synergism bioassays were repeated on two to four separate treatment days with three to five colonies from each stock.

### Bee Age Bioassays

Because of the division of labor in honey bees, younger bees typically perform tasks within the hive, while older bees forage [17]. To obtain a population of bees that could be nurses or foragers at each time in our collection schedule (3, 14, 28, and 42 days of age), we used single-cohort colonies (SCC, [22, 23]) of Italian bees. These SCCs were created by housing approximately 2500 newly eclosed adult bees from our Italian colonies in a nucleus box with a naturally mated Italian queen, 2 combs of pollen and honey, and 2 empty combs to allow egg laying. To indicate age, the dorsal thorax of each bee was marked with a dot of paint (Testors, Rockford, IL) prior to colony placement at the USDA-ARS apiary. Each SCC was housed in the laboratory in a dark incubator at 33 ± 0.1°C with 70 ± 5% humidity for 5 days to allow the queen to begin egg laying and adult bee maturation. Four SCCs were set up on 24-June 2014, 25-June–2014, 25-July–2014, and 26-July–2014.

To test the effect of age on insecticide sensitivity, a mix of nurses and foragers were collected from SCCs at 3, 14, 28, and 42 days of age using forceps or an insect vacuum (Insect Aside, Farmington WA). Bees were placed in groups of 20 into 475-ml wax cups (Innopack, Orange, CA) and then weighed. Bees were bioassayed as described below on the day they were collected.

### Insecticides and Bioassays

We evaluated the sensitivity of honey bees to nine pesticides including two neonicotinoids: thiamethoxam and imidacloprid, three organophosphates: malathion, naled, and coumaphos (as a synergist); three pyrethroids: resmethrin, etofenprox and phenothrin; the formamidine amitraz (as a synergist), and the P450 inhibitor piperonyl butoxide (PBO, as a synergist). All materials were of >98% purity and purchased from ChemService (West Chester, PA). Stock solutions of each compound were dissolved in acetone.

Stock solutions were diluted to include ≥4 concentrations that provided >0% and <100% mortality. Cups of bees were randomly assigned to each treatment. Bioassays between stocks with the same insecticide were performed on the same treatment day. Topical bioassays with malathion, naled, resmethrin, etofenprox, and phenothrin were performed by applying a 1 uL drop of insecticide (in acetone) with a mechanical Hamilton syringe (Reno, NV) to the thoracic notum of a bee anesthetized with CO_2_ for less than 1 minute. Controls received 1 uL of acetone only. Bees were fed via 50% sucrose soaked cotton balls after treatment. Topical bioassays were reported in units of ng insecticide/mg bee. Imidacloprid and thiamethoxam toxicity were assessed with a feeding bioassay performed by filling a perforated 1.5 ml microcentrifuge tube with 1 ml of 50% sucrose solution containing either imidacloprid or thiamethoxam, which was inserted through the tulle cup cover. Control feeding bioassays were conducted with 50% sucrose solution with 0.001% acetone. The volume of sucrose solution consumed was not recorded so toxicity values are reported in units of ng insecticide/mL sucrose solution/mg bee. Bees were held at the environmental conditions listed above.

Synergism studies were performed with the experimentally determined maximum sublethal dose of PBO (75 ug/bee), amitraz (0.5 ug/bee), or coumaphos (5 ug/bee) by applying 1 uL of the synergist to the thoracic notum of each bee one hour before treatment with phenothrin. The same sublethal dose of each synergist was used for all stocks. Controls were pretreated with synergist then treated with acetone. Synergism tests were performed alongside bioassays with phenothrin alone with bees from the same cohort and experimental conditions. The Synergism Ratio (SR) was calculated by dividing the LD_50_ of phenothrin alone by the LD_50_ of phenothrin from insects pretreated with synergist. Bees were held at environmental conditions listed above.

Mortality in all bioassays was unblinded and recorded at 24 hours after insecticide application because either there was no increase in mortality at 48 or 72 hours or the small increase in mortality at 48 and 72 hours did not change the LD_50_/LC_50_ values or slope of the dose-response curve. Individuals that were ataxic or unable to right themselves were scored as dead. Bioassays to test for sensitivity between stocks and synergistic interactions were repeated on two to four separate treatment days with three to five colonies from each stock. Aged bee bioassays were replicated three times with bees from four SCCs established on two independent dates.

The LD_50_/LC_50_ values were calculated using probit analysis with Abbott’s correction for control mortality [24] and standardized by body weight (Minitab, State College, PA). Toxicity was considered significantly different if the 95% confidence intervals of the LD_50_ or LD_50_ values did not overlap.

## Results

### Stock Weights

The weight of bees varied significantly within and between stocks. Bees from Italian colonies used in our studies ranged from 73.7 to 86.0 mg, representing a 14.3% variation in weight from colony to colony. Italian bees were the smallest of the three stocks we compared with an average weight of 82.0 ± 1.3 mg compared to an average weight of 87.5 ± 1.5 mg and 93.7 ± 1.9 mg for Carniolan and Russian bees, respectively. The weights among stocks were all significantly different (One-Way ANOVA, F = 20.88, P<0.001).

### Insecticide Sensitivity of Honey Bee Stocks

There was variation in insecticide susceptibility among genotype, and variation was insecticide dependent (Tables 1 and 2). Malathion sensitivity was least in Italian bees, intermediate in Carniolan bees and greatest in Russian bees, although LD_50_s differed only by 0.07 ng/mg bee among the stocks. There were no differences among stocks in sensitivity to naled or etofenprox. Russian bees were less sensitive to phenothrin compared to Carniolan and Italian bees, which were equally sensitive. Russian and Carniolan bees were 1.7- and 2.9-fold less sensitive to resmethrin than Italian bees, respectively. The largest difference in sensitivity among stocks was for imidacloprid. Italian bees were very sensitive to imidacloprid, whereas Russian and Carniolan bees were 15.7- and 33.3-fold less sensitive than Italian bees, respectively. Differences in sensitivity were smaller for thiamethoxam. Carniolan and Russian bees were 1.4- and 3.4-fold less sensitive than Italian bees, respectively, while being significantly different from each other.

**Table 1:** Variation in organophosphate and pyrethroid sensitivities by topical bioassays among three stocks of commonly used honey bees. Different letters for LD_50_ values in the same row indicate significant differences. Different symbols for slope values in the same row indicate significant differences. The LD_50_ values are in units of ng insecticide/mg bee.

	Carniolan			Italian			Russian		
Compound	n	LD_50_ (95% CI)	Slope (SE)	n	LD_50_ (95% CI)	Slope (SE)	n	LD_50_ (95% CI)	Slope (SE)
Malathion	484	1.10 (1.07–1.14)^ab^	10.9 (0.9)^‡^	1175	1.12 (1.10–1.14)^a^	10.8 (0.6)^‡^	838	1.05 (1.03–1.09)^b^	9.3 (0.6)^‡^
Naled	604	0.54 (0.51–0.56)^a^	6.5 (0.5)^‡^	910	0.52 (0.50–0.53)^a^	8.4 (0.5)*	910	0.53 (0.51–0.54)^a^	8.6 (0.5)*
Etofenprox	647	0.77 (0.69–0.85)^a^	2.6 (0.2)^‡^	404	0.92 (0.78–1.05)^a^	2.7 (0.3)^‡^	410	0.79 (0.74–0.84)^a^	5.2 (0.6)*
Phenothrin	717	0.84 (0.81–0.87)^b^	7.2 (0.6)^‡^	1372	0.80 (0.77–0.83)^b^	5.3 (0.4)*	747	1.06 (1.00–1.12)^a^	4.5 (0.3)*
Resmethrin	1146	0.58 (0.55–0.60)^c^	4.4 (0.2)^‡^	857	0.20 (0.17–0.22)^a^	3.1 (0.2)*	972	0.34 (0.32–0.35)^b^	6.8 (0.4)^$^

**Table 2:** Variation in neonicotinoid sensitivity by feeding bioassays among three stocks of commonly used honey bees. Different letters for LC_50_ values in the same row indicate significant differences. Different symbols for slope values in the same row indicate significant differences. The LC_50_ values for imidacloprid and thiamethoxam are expressed in ng/ml/mg bee.

	Carniolan			Italian			Russian		
Compound	n	LC_50_ (95% CI)	Slope (SE)	n	LC_50_ (95% CI)	Slope (SE)	n	LC_50_ (95% CI)	Slope (SE)
Imidacloprid	302	83.3 (59.1–148.2)^c^	1.1 (0.2)^‡^	590	2.5 (0.6–5.0)^a^	0.9 (0.2)^‡^	720	39.3 (32.6–47.5)^b^	1.3 (0.1)^‡^
Thiamethoxam	778	2.70 (2.50–2.90)^b^	4.2 (0.3)^‡^	257	1.86 (1.46–2.32)^a^	2.0 (0.2)*	169	6.34 (4.08–8.64)^c^	1.8 (0.3)*

The slopes of the log dose probit lines from bioassays among stocks were significantly different in most cases ((t-test, p<0.05; Tables 1 and 2). Slopes for all three stocks in the resmethrin bioassay were significantly different. The slopes for Carniolan bees were significantly different compared to Italian and Russian bees in phenothrin and naled bioassays. The slope of the log dose probit lines for etofenprox was significantly different for Italian and Carniolan bees compared to Russian bees. There were no significant differences in the slopes of the log dose probit lines for malathion or imidacloprid.

### Synergist Bioassays

Pretreatment with amitraz, coumaphos, or PBO significantly increased phenothrin sensitivity in all honey bee stocks (Table 3). Honey bee genotype affected sensitivity to phenothrin after exposure to a synergist. Whereas coumaphos synergism varied from 5- to 7-fold across stocks, synergized LD_50_ values were the same across stocks. Pretreatment with amitraz increased phenothrin sensitivity in Italian bees 1.4-fold, but increased phenothrin sensitivity in Russian bees 2.9-fold. Italians had the lowest synergism by PBO (2.5-fold) which was significantly lower than that measured with Carniolan (3.6-fold) and Russian bees (4.5-fold).

**Table 3:** Miticides and P450 inhibitor synergizes phenothrin sensitivity among three stocks of honey bees. All Synergism Ratios (SR) indicate significantly increased sensitivity compared to non-synergized phenothrin treatment (Table 1). Different letters for LD_50_ values in the same row indicate significant differences. Different symbols for slope values in the same row indicate significant differences. The LD_50_ values are in units of ng phenothrin/mg bee.

	Carniolan				Italian				Russian			
Synergist	n	LD_50_ (95% CI)	Slope (SE)	SR	n	LD_50_ (95% CI)	Slope (SE)	SR	n	LD_50_ (95% CI)	Slope (SE)	SR
Amitraz	419	0.36 (0.31–0.39)^b^	4.1 (0.5)^‡^	2.4	567	0.56 (0.52–0.60)^a^	3.7 (0.4)^‡^	1.4	386	0.36 (0.26–0.43)^b^	2.3 (0.5)*	2.9
Coumaphos	229	0.14 (0.12–0.16)^a^	4.4 (0.5)^‡^	6.0	207	0.16 (0.13–0.21)^a^	3.0 (0.5)*	5.0	292	0.15 (0.14–0.17)^a^	7.8 (0.9)^$^	7.0
PBO	316	0.24 (0.20–0.27)^b^	3.4 (0.4)^‡^	3.6	628	0.32 (0.29–0.35)^a^	3.2 (0.3)^‡^	2.5	389	0.24 (0.21–0.26)^b^	3.2 (0.3)^‡^	4.5

The slope of each synergized phenothrin response curve was significantly reduced compared to the unsynergized slope in all instances except in the case of coumaphos synergism in Russian bees where the slope was increased (t-test, p<0.05; Table 3). The slopes of the log dose probit lines from phenothrin bioassays synergized with coumaphos were significantly different among stocks. The slope of the log dose probit lines of phenothrin bioassays synergized with amitraz was significantly different for Carniolan and Italian bees compared to Russian bees. There was no difference in the phenothrin bioassay slopes among stocks when synergized with PBO.

### Phenothrin and Naled Bioassays on Aged Bees

**Fig 1:**
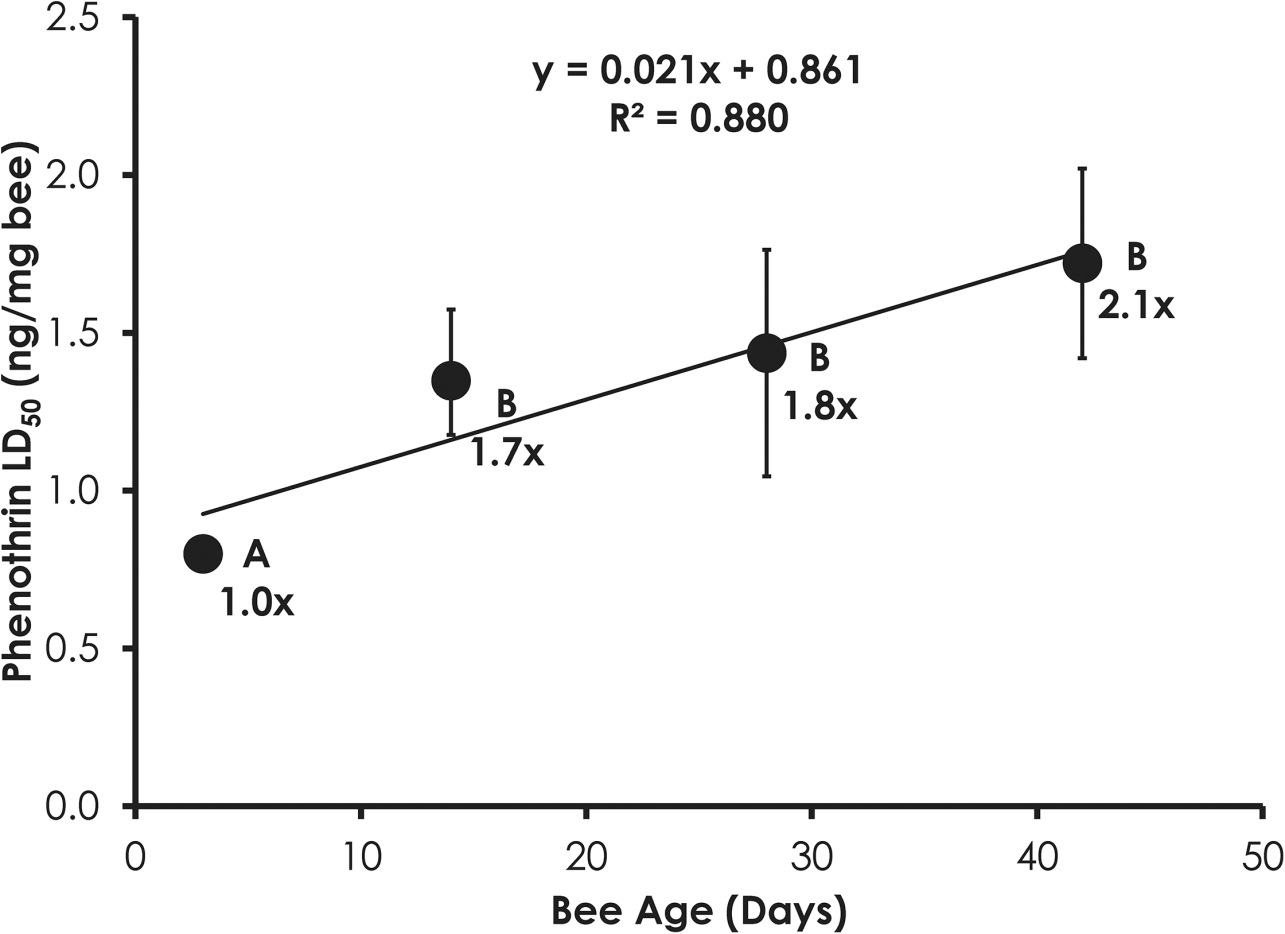
Phenothrin sensitivity decreases with age in honey bees. Letters above data points indicate significant differences in the LD_50_ values.

**Fig 2:**
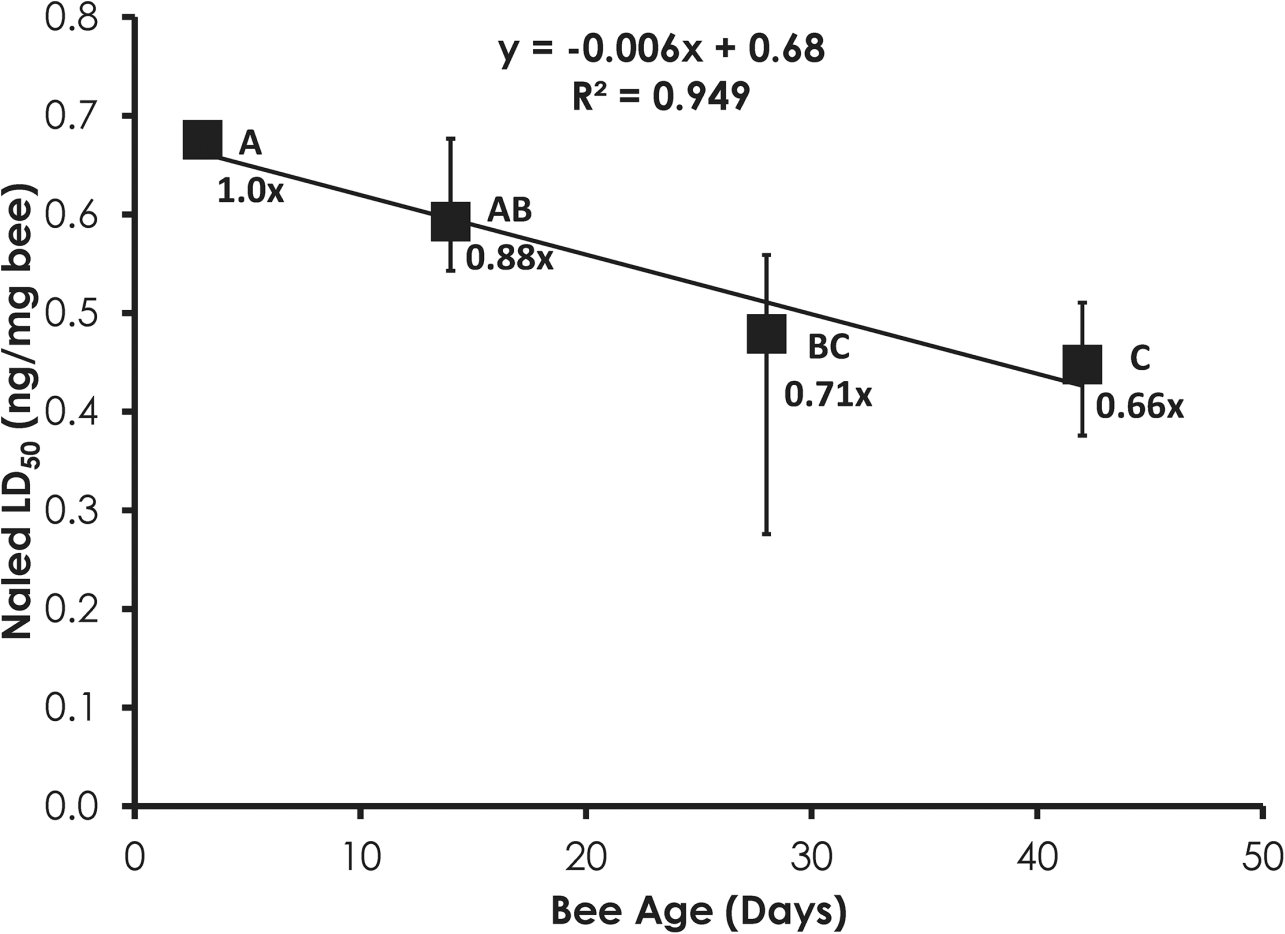
Naled sensitivity increases with age in honey bees. Letters above data points indicate significant differences in the LD_50_ values.

Sensitivity to phenothrin and naled was significantly correlated with age. In the case of phenothrin, 3-day-old bees were significantly more sensitive than 14-, 28-, and 42-day-old bees (Fig 1). Phenothrin sensitivity significantly decreased by 1.7-, 1.8-, and 2.1-fold in 14-, 28-, and 42-day-old bees, respectively, compared to 3-day-old bees. The decrease in phenothrin sensitivity was significantly correlated with age (Linear Regression, F = 71.13, p = 0.014, R^2^ = 0.880). In contrast, naled sensitivity significantly increased with age (F = 27.23, p = 0.035; R^2^ = 0.949, Fig 2). The increase in naled sensitivity continued as bees aged as 28-day-old bees were significantly more sensitive than 3-day-old bees while 42-day-old bees were significantly more sensitive than both 3-and 14-day-old bees.

The slopes of both phenothrin and naled dose response curves became reduced as bees aged. For phenothrin, the slope of the dose response curve was significantly steeper for 3-day-old bees compared to 14- (t = 3.35, df = 5, p = 0.02) and 42-day-old bees (t = 4.41, df = 5, p = 0.006). The slopes of the naled dose response curve was significantly reduced for 3-day-old bees compared to 14- (t = 2.81, df = 4, p = 0.048) 28- (t = 3.16, df = 4, p = 0.034) and 42-day-old bees (t = 5.69, df = 4, p = 0.005).

## Discussion

Our results show that genetics, age, and synergists are significant sources of variation that can result in different responses to insecticides. Using an average LD_50_ across all stocks, there was a 3-fold range in the sensitivity of topically applied insecticides with resmethrin being the most toxic, and malathion being the least toxic. This difference in the relative toxicity of resmethrin and malathion is much less than a 20-fold difference previously reported, mostly due to differences in malathion sensitivity [25]. Sensitivity differences among stocks of bees have rarely been reported in the literature, but most studies demonstrate that Italian bees are the most sensitive stock of honey bee. African bees (*Apis mellifera scutellata*) were approximately 2-fold less sensitive to azinphosmethyl, methyl parathion, and permethrin, but 2-fold more sensitive to carbaryl compared to Italian bees [26]. Cyfluthrin was 3-fold more toxic to African bees than European bees (likely Italians) [27]. Italian bees were more sensitive than Carniolan and German bees (*Apis mellifera mellifera*) to the neonicotinoids imidacloprid and thiamethoxam [28]. The differences in sensitivity to imidacloprid and thiamethoxam in that study were 5- and 3-fold, respectively. The relative toxicity values in our study were much higher for imidacloprid (15- to 33-fold), but largely in agreement with those for thiamethoxam (1.3- to 3.4-fold). Nonetheless, no differences in imidacloprid sensitivity were reported between German and Caucasian bees [29]. However, these studies did not consider bee weight as a confounding factor in insecticide sensitivity. Therefore, any comparisons between studies should be made with reservations. Because of the drastic difference in insecticide sensitivity in this study and others, identifying the stock and size of bees used in toxicology studies is advisable so that valid comparisons may be made among these studies.

Besides the differences in LD_50_/LC_50_ values, the slopes of the dose response curves were significantly different between stocks in many instances. The slope of the dose response curves can indicate the potency of an insecticide, the dose range that generates a response in an organism, and the degree of genetic homogeneity involved in insecticide interactions (i.e. genes for detoxification enzymes and target sites) in the test population [30]. Differences in the slopes of the log dose probit lines can yield dramatic differences at the extremes of the dose response curve. For example, the LD_50_ for resmethrin was 1.7-fold higher in Russian bees compared to Italian bees. However, due to the dramatic differences in slopes between these two stocks, the LD_10_ dose was 2.8-fold higher for Russian bees than Italian bees, but there was no difference in the LD_90_ dose between both stocks. These differences in slopes are important considerations when distinguishing differential insecticide sensitivity between stocks because the exaggerated differences at the low end of the dose response curve mimic field conditions where honey bees are typically exposed to doses and concentrations below the LD_50_ value of insecticides [6]. Much research exists on the detrimental impacts of insecticides at doses or concentrations below the LD_50_ value on honey bee learning, foraging, development and longevity [10, 31, 32].

The magnitude of the differences in insecticide sensitivity among stocks appears to be a function of the class of insecticide tested. The small differences in malathion sensitivity between Italian and Russian bees was likely biologically irrelevant as the overlap in the 95% CI of the LD_50_ values only differed by 0.01 ng/mg bee. The negligible differences in the LD_50_ and steep slopes of the organophosphate bioassays suggest that the detoxification pathways (i.e., esterases) and target site sensitivities (i.e., acetylcholine esterases) are homogenous across and within stocks. Whereas malathion was the least toxic topically applied insecticide, it demonstrated the steepest dose-response slope. Therefore, precise dilutions of malathion-containing products are of utmost importance because a slight variation in concentration may result in dramatic differences in honey bee toxicity.

The significant differences in LD_50_s and slopes of pyrethroid bioassays indicate large genetic variance in pyrethroid sensitivity among and within stocks [30]. Detoxification of pyrethroids by P450s is likely to be responsible for the variation in pyrethroid toxicity because inhibition of P450 metabolism by pretreatment with PBO resulted in differential phenothrin sensitivity (Table 3 and [33]). The variation in the degree of phenothrin synergism with PBO demonstrates varying detoxification capabilities between stocks. Further bioassays with other synergists to inhibit esterases (i.e. S,S,S-tributyl phosphorotrithionate (DEF)) as well as molecular investigations on the occurrence of mutations in the voltage-gated sodium channel associated with pyrethroid resistance [34] would assist in accurately identifying the biochemical and physiological mechanisms underlying these differences.

The largest variation in insecticide sensitivity was seen in neonicotinoid bioassays as evident in the 34-fold difference in imidacloprid sensitivity between Italian and Carniolan bees. In addition to determining pyrethroid toxicity as mentioned above, P450s are capable of detoxifying imidacloprid and other neonicotinoids [35, 36]. The large variation in sensitivity and slope suggests that there may be differences in the nicotinic acetylcholine receptors (nAChRs) that are the target site of neonicotinoids [37]. The honey bee genome contains 11 nAChR subunit genes [38] and mutations as well as post-transcriptional modifications of these genes can affect neonicotinoid sensitivity [39, 40]. Therefore, future investigations in the molecular mechanisms that underlie these dramatic differences in imidacloprid sensitivity between stocks should be productive.

Synergism of phenothrin toxicity among stocks with PBO, amitraz, and coumaphos was expected because synergism studies with another pyrethroid (tau-fluvalinate) produces similar interactions [7]. The results of our synergism studies demonstrate that synergists affect these stocks differentially. The significant increase in phenothrin sensitivity with PBO pretreatment suggests that P450s are involved in the detoxification of phenothrin, which is consistent with previous work on other pyrethroids in honey bees [41, 42]. The extent of PBO synergism was less in Italian bees compared to Carniolan and Russian bees, suggesting the detoxification capacity of P450s is reduced in Italian bees, potentially contributing to the high sensitivity of that stock to most insecticides. The significant variation in PBO synergism demonstrates that the P450-mediated detoxification capacity varies among stocks. This suggests that differences in P450 expression among stocks may contribute to heightened insecticide sensitivity rather than a deficit of detoxification genes encoded in the honey bee genome [43].

Phenothrin synergism with amitraz is more nuanced as amitraz acts primarily at octopamine receptors [44]. However, amitraz and other formamidine insecticides may promote cooperative binding of pyrethroids at the voltage-gated sodium channels, thus increasing target-site sensitivity [45]. The exact mechanism of reduced amitraz synergism in Italian bees compared to Carniolan and Russian bees is unclear, but these results suggest that there are genetic differences between stocks that manifest in variable synergism.

Coumaphos provided the greatest phenothrin synergism and equalized phenothrin sensitivity across stocks. Synergism of pyrethroids with coumaphos has been documented, and our synergism levels are consistent with reported values [7, 8]. It is worth noting that the sublethal doses of amitraz (0.5 ug) and coumaphos (5 ug) we used in our synergism bioassays were near the maximum doses bees may encounter in the colony [8, 46]. Therefore, the doses used in these synergism tests are relevant for field exposure. These data underscore the detrimental impacts that beekeeper-applied miticides (especially when over applied) may have on the sensitivity of insecticides encountered outside of the colony and emphasize the value of non-chemical mite control.

The decreased phenothrin sensitivity and increased naled sensitivity that was highly correlated with age demonstrated that age-dependent transitions in physiology are significant factors in determining insecticide sensitivity. Honey bee metabolism changes dramatically with age coincident with behavioral changes. At the onset of flight in forager bees, metabolic rate increases nearly 100-fold [18, 19, 47]. Among these physiological transitions with age, oxidase specific activity by P450s increases with age [48, 49]. P450s detoxify pyrethroids via hydroxylation [33, 50]. Therefore, the decreased phenothrin sensitivity with age is corroborated by increased P450 activity with age. Naled is detoxified by esterases [51] and the relationship of esterase activity with bee age is unknown. Therefore, future studies to determine esterase activity levels with bee age may provide a plausible physiological mechanism for decreased naled sensitivity. Older forager bees spend most of their time outside of the protective confines of the colony and likely experience higher pesticide exposure relative to their in-hive nest mates. The advantage of using young bees as we did is that we could control for nutrition, physiological status, behavioral state, and environmental conditions. Due to these age differences in insecticide sensitivity, caution should be exercised when comparing data from bees of unknown age and extrapolating results from young bees to old bees and vice-versa.

In conclusion, we have demonstrated that there is sufficient genetic variation in commercial stocks of honey bees to result in different levels of insecticide sensitivity and the magnitude of these differences was mostly dependent on the class of insecticide applied. Italian bees were the most sensitive of the three stocks we tested and future experiments on risk assessment may benefit from using this uniquely sensitive stock to ensure a high level of safety in projecting hazards to honey bees. Synergism tests suggest that metabolic capacity varies among stocks and may underlie the particular sensitivity of the Italian bees. Also, pretreatment with amitraz or coumaphos increased phenothrin sensitivity demonstrating the potential for miticides to affect honey bee health regardless of genetic background. The changes in insecticide sensitivity with age are very important to note as older bees are typically the ones most exposed to these insecticides as foragers. These data provide a sound foundation on which best management practices in agriculture and public health, environmental policies, and trait selection for bee-keepers can be used in an effort to minimize non-target impacts of insecticides on honey bees.

## Supporting Information

S1 Dataset Individual bee weight of commonly used stocks of honey bees and weight of Italian bees at 3, 14, 28, and 42 day intervals. (XLSX)

S2 Dataset Bioassay data from three commonly used stocks of honey bees and aged Italian bees. (XLSX)

S3 Dataset Mortality of Italian honey bees against resmethrin at 1, 2, and 3 days. (PDF)
