# Supplementary figures and images for: Genetics, Synergists, and Age Affect Insecticide Sensitivity of the Honey Bee, Apis mellifera
Source: PLoS One. 2015 Oct 2;10(10):e0139841. doi: 10.1371/journal.pone.0139841 (PMC4592006; doi:10.1371/journal.pone.0139841)

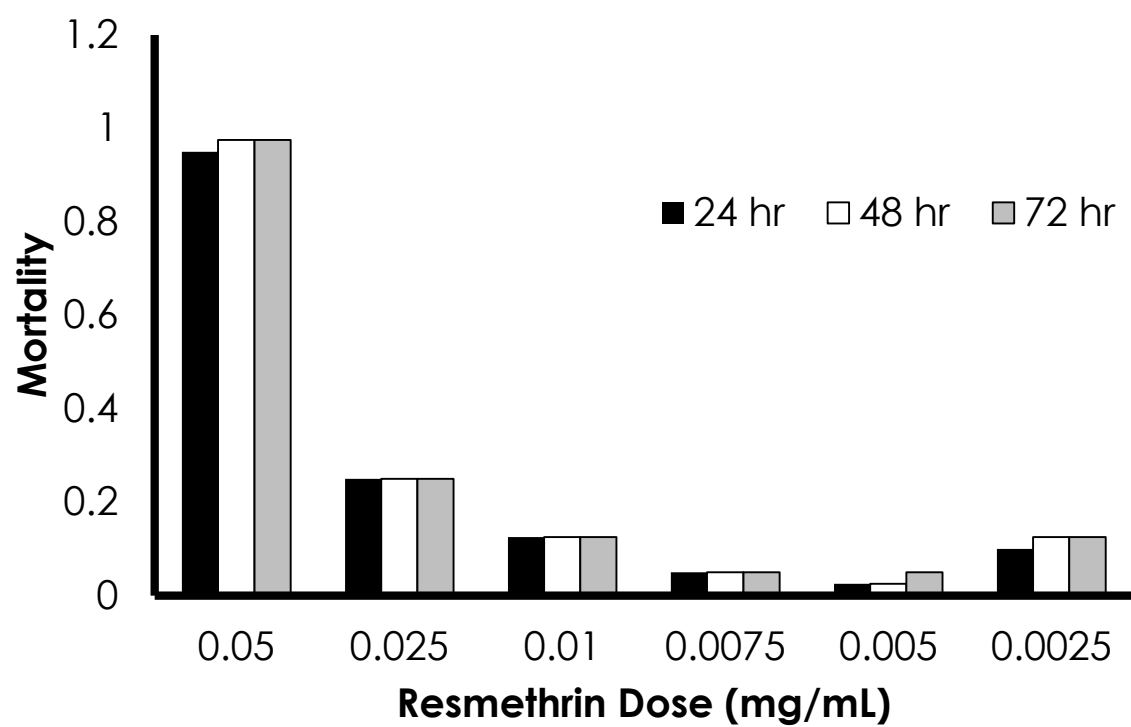

Supplement: S3 Dataset — (PDF) [file pone.0139841.s003.pdf]
